# Reply: Interaction or mediation by adult obesity of the relation between fetal famine exposure and type 2 diabetes?

**DOI:** 10.1093/ije/dyy295

**Published:** 2019-01-21

**Authors:** Ruogu Meng, Jun Lv, Liming Li

**Affiliations:** 1 Department of Epidemiology and Biostatistics, School of Public Health, Peking University Health Science Center, Beijing, China; 2 Peking University Institute of Environmental Medicine, Beijing, China

We thank Li and Lumey for their interest in our study. As mentioned by Li and Lumey, the associations of fetal exposure to famine with adult obesity and diabetes risks have been observed in Dutch famine studies. Also, there is a well-established association between obesity and diabetes. After the combination of non-exposed and early-childhood-exposed participants as the reference, our study reported the similar findings that both fetal exposure to the Chinese Great Famine and adult overweight or obesity were associated with increased risks of type 2 diabetes.^1^ However, the relations between famine exposure, adult obese status and diabetes risk do not necessarily lead to an interaction between the effects of famine exposure and adult obesity on diabetes risk. Similarly, two previous studies also did not find that general obesity during adulthood interacts with the Chinese famine experience in early life to increase the risk of type 2 diabetes.^2^^,^^3^ To the best of our knowledge, our study is the first to investigate the effect of the interaction of fetal famine exposure with abdominal obesity on diabetes risk.

In Li and Lumey's presented analysis, compared with non-exposed and early-childhood-exposed participants, participants with general or abdominal obesity had a higher risk of adulthood diabetes associated with fetal exposure to famine than the non-obese participants. The test for relative excess risks due to interaction (RERI) was statistically significant for both general and abdominal obesity.

We repeated the analysis as done by Li and Lumey (Table 1). The association between fetal famine exposure and diabetes was consistent across subgroups according to the body mass index (BMI; *P*_interaction_ = 0.181) but different across subgroups according to the waist-to-hip ratio (WHR; *P*_interaction_ = 0.018). The RERI, calculated as introduced by Vanderweele *et al*. using Statistical Analysis System (SAS),^4^ was 0.01 [95% confidence interval (CI): −0.03, 0.05] (*P* for additive interaction = 0.588) for BMI and 0.13 (95% CI: 0.09, 0.17) (*P* for additive interaction < 0.001) for WHR. The present results are basically the same as our previous ones,^1^ but inconsistent with Li and Lumey's analyses based on basic tabulations.

**Table 1.** Multivariable-adjusted HRs (95% CIs) for association between famine exposure in early life and type 2 diabetes according to adult obesity measures among 88 830 participants

	Cases	Case/PYs (1000)	Non- and early-childhood exposed	Fetal exposed	*P*_interaction_
BMI at baseline, kg/m^2^					0.181
<24.0	431	1.2	1.00	1.12 (0.84, 1.48)	
≥24.0	941	3.2	1.00	1.32 (1.10, 1.58)	
WHR at baseline					0.018
Men <0.90, women <0.85	351	1.2	1.00	0.98 (0.72, 1.34)	
Men ≥0.90, women ≥0.85	1021	2.9	1.00	1.35 (1.13, 1.61)	

The multivariable model was adjusted for age (years), sex (men or women), education (no formal school, primary school, middle school, high school, college, or university or higher), marital status (married, widowed, divorced or separated, or never married), smoking (never smoker, former smoker who quit for reasons other than illness, current smoker or former smoker who quit because of illness: 1–14, 15–24, or ≥25 cigarettes/day), alcohol consumption (non-weekly drinker, former weekly drinker, weekly drinker, daily drinker: <15, 15–29, 30–59 or ≥60 g/day), physical activity (MET-hour/day), intake of fruits, vegetables, red meat, white rice and wheat (day/week; calculated by assigning participants to the mid-point of their consumption category), and family history of diabetes (yes or no). Analysis of BMI was further adjusted for WHR (men: <0.90, 0.90–0.94 or ≥0.95; women: <0.85, 0.85–0.89 or ≥0.90). Analysis of WHR was further adjusted for BMI (<18.5, 18.5–23.9, 24.0–27.9 or ≥28.0).

HR, hazard ratio; CI, confidence interval; PYs, person-years; BMI, body mass index; WHR, waist-to-hip ratio; MET, metabolic equivalent of task.

Table 2 presents the results of stepwise adjusted models for the association between fetal famine exposure and diabetes. Further adjustment for BMI and WHR led to a trivial change in the hazard ratio estimate. The difference method is one of the traditional approaches to mediation analysis.^5^ An apparent reduction in the exposure coefficient, when comparing the model without the mediator with that adding the mediator, is thought to be indicative of mediation because the mediator seems to explain some of the effects of the exposure on the outcome. We, therefore, explain our results as that adult obesity might not be a mediator of the association.

**Table 2.** HRs (95% CIs) for associations between famine exposure in early life and type 2 diabetes among 88 830 participants

	Non- and early- childhood exposed	Fetal exposed
Case	1054	318
Case/PYs (1000)	2.1	2.3
Age-, sex-adjusted	1.00	1.27 (1.09, 1.48)
Multivariable-adjusted	1.00	1.27 (1.09, 1.48)
Further adjusted for BMI and WHR	1.00	1.25 (1.07, 1.45)

Multivariable model was adjusted for age (years), sex (men or women), education (no formal school, primary school, middle school, high school, college, or university or higher), marital status (married, widowed, divorced or separated, or never married), smoking (never smoker, former smoker who quit for reasons other than illness, current smoker or former smoker who quit because of illness: 1–14, 15–24, or ≥25 cigarettes/day), alcohol consumption (non-weekly drinker, former weekly drinker, weekly drinker, daily drinker: <15, 15–29, 30–59, or ≥60 g/day), physical activity (MET-hour/day), intake of fruits, vegetables, red meat, white rice and wheat (day/week; calculated by assigning participants to the mid-point of their consumption category), and family history of diabetes (yes or no). Further adjusted for BMI (<18.5, 18.5–23.9, 24.0–27.9 or ≥28.0) and WHR (men: <0.90, 0.90–0.94 or ≥0.95; women: <0.85, 0.85–0.89 or ≥0.90).

HR, hazard ratio; CI, confidence interval; PYs, person-years; BMI, body mass index; WHR, waist-to-hip ratio; MET, metabolic equivalent of task.

As suggested by Li and Lumey, significantly elevated diabetes risk associated with fetal famine exposure in obese rather than in non-obese participants indicated that adult obesity is a mediator of the famine–diabetes association. However, if obesity is a mediator on a causal path from fetal famine experience to adult diabetes, the association between fetal famine exposure and diabetes tends to be attenuated towards the null in both subgroups of obesity measure.^6^

## Funding

This work was supported by grants (81373082, 81390544) from the National Natural Science Foundation of China. The CKB baseline survey and the first re-survey were supported by a grant from the Kadoorie Charitable Foundation in Hong Kong. The long-term follow-up is supported by grants from the UK Wellcome Trust (088158/Z/09/Z, 104085/Z/14/Z); by a grant from the Chinese Ministry of Science and Technology (2011BAI09B01). Dr Lv is supported by the State Scholarship Fund of China Scholarship Council (201506015053). The funders had no role in the study design, data collection, data analysis and interpretation, writing of the report, or the decision to submit the article for publication.
